# Perspectives of Cancer Survivors with Low Income: A Content Analysis Exploring Concerns, Positive Experiences, and Suggestions for Improvement in Survivorship Care

**DOI:** 10.3390/curroncol30090590

**Published:** 2023-09-01

**Authors:** Irene Nicoll, Gina Lockwood, Margaret I. Fitch

**Affiliations:** 1 Health Care Consultant, Toronto, ON M4C 4V9, Canada; nicollirene4@gmail.com; 2 Biostatistician Consultant, Toronto, ON M4C 4V9, Canada; lockwo12@gmail.com; 3 Faculty of Nursing, University of Toronto, 207 Chisholm Ave., Toronto, ON M4C 4V9, Canada

**Keywords:** cancer, low income, qualitative analysis, cancer survivor perspectives

## Abstract

The number of cancer survivors in Canada has reached 1.5 million and is expected to grow. It is important to understand cancer survivors’ perspectives about the challenges they face after treatment is completed. Many factors create barriers to accessing assistance, and limited income may be a significant one. This study is a secondary analysis of data from a publicly available databank (Cancer Survivor Transitions Study) regarding the experiences of Canadian cancer survivors. The goal was to explore major challenges, positive experiences, and suggestions for improvement in survivorship care for low-income Canadian cancer survivors one to three years following treatment. A total of 1708 survey respondents indicated a low annual household income (<$25,000 CD). A content analysis was performed utilizing written comments to open-ended questions. The major challenges respondents described focused on physical capacity limits and treatment side effects; positive experiences emphasized support and attentive care; and suggestions for improvements highlighted the need for better support, information about self-care and side effect management, and timely follow-up care. The relationships between household income and the management of survivors’ physical, emotional, and practical concerns require consideration. The design of follow-up care plans, programs, services, and financial assessments of patients may prepare survivors for predictable issues and costs in their transition to survivorship.

## 1. Introduction

Advances in cancer screening and treatment have resulted in a growing number of cancer survivors, which is expected to exceed 25 million worldwide by 2025 [1,2]. A cancer diagnosis has physical, emotional, and practical consequences for the individual who is diagnosed as well as their family members [3]. These consequences can continue after the completion of cancer treatment for some individuals, influencing their quality of life and well-being [4,5,6]. Over 80% of Canadian cancer survivors have reported living with ongoing challenges after the completion of their cancer treatment and over 60% had difficulty accessing assistance for these concerns [7].

One of the socioeconomic factors that can act as a barrier for individuals in accessing health care in general, and specifically cancer care, is financial [8]. Income level has been identified as a barrier to being screened for cancer [9], diagnosed and treated for the disease [10], and attending mental health [11] and rehabilitation services [1]. Additionally, financial toxicity, or problems individuals face related to the cost of medical care, has been identified as a side effect of cancer treatment, occurring not only during treatment but continuing after treatment finishes [12,13,14]. The impact of financial toxicity occurs for the person with cancer and for their family members [15]. For those who are already in a low-income bracket, being diagnosed with cancer can be highly distressing and have long-term implications [16,17]. 

Research regarding the financial burden for Canadian cancer patients undergoing treatment has shown that higher proportions of those with incomes below $50,000 CD experienced financial hardship compared to those with a higher income [18]. However, little work has focused on financial hardship following the end of cancer treatment in this country. Some work regarding the experiences of Canadian cancer survivors identified those with low incomes (<$25,000 CD) reported more physical, emotional, and practical concerns and difficulty accessing a range of health-related services following the completion of their cancer treatment than cancer survivors in higher income categories [19,20,21]. Additional work is necessary to gain insight into the influences of these experiences.

These observations come from within a healthcare system described as having universal healthcare coverage. However, the coverage is only for “medically necessary care”, and not all aspects of care are fully funded. Healthcare is under provincial jurisdiction, and policy regarding specific coverage varies from province to province. For example, coverage for home care has monthly limits that vary across the country, some diagnostic tests and procedures are not covered, and certain ambulatory drugs are not covered. Policies and programs assisting with income replacement and means-based medical care also vary by province. At present, 6.4% of the Canadian population is reported to be living in poverty and trends show a growing number are dealing with healthcare challenges, unmet housing needs, and food insecurity [22]. In addition, poverty statistics exclude people living in “hidden poverty”, when individuals earn above the poverty line but cannot afford adequate food, housing, childcare, or other basic necessities [23].

Understanding the perspectives of cancer survivors who have a low household income level is important for designing appropriate approaches to survivorship care that are tailored for these individuals. The current study is a qualitative analysis of the open-ended survey responses from respondents who reported incomes <$25,000 CD on the Canadian Transition Study. The Canadian Transition Study, conducted in 2016, created a national, publicly available database containing information about the experiences of cancer survivors. 

## 2. Methods

The need for Canadian data regarding survivors’ perspectives to inform cancer system developments in survivorship care led to the Canadian Partnership Against Cancer conducting the Transitions Survey [7]. This pan-Canadian survey was distributed across 10 provinces to a randomly selected cancer registry sample of 40,790 cancer survivors between one and three years following the completion of their cancer treatment [7]. It was designed for cancer survivors most apt to be followed in the community, to identify their needs, and explore their experiences with follow-up care. The period of one to three years allowed survivors to have experienced follow-up care. Eligibility included adult survivors over the age of 35 years old with breast, prostate, colorectal, and melanoma diseases with no metastatic spread and selected hematological cancers (e.g., Hodgkin’s Lymphoma, diffuse B cell lymphoma, acute myelogenous leukemia, acute lymphocytic leukemia); and those aged 18 to 34 years, with all non-metastatic cancer types, except testes, where metastatic disease was included because of its high survival rate. Ethics approval was granted by the respective ethics boards in each provincial cancer agency and participants signed their consent prior to participation. The detailed report of this survey was previously published [7]. The database created from this survey is publicly available and allows access by researchers interested in exploring perspectives of Canadian survivors. 

This paper reports a secondary analysis of selected items from the Transitions Study database. It highlights a content analysis of written responses from respondents who indicated an annual household income of <$25,000 CD, and responded to open-ended questions about the biggest challenge they had faced since completing cancer treatment, their positive experiences during care, and their suggestions for improvements. The first question was intended to understand what survivors found most troublesome or difficult during the early survivorship period. The second question was intended to understand what they valued or appreciated about survivorship care. The third question focused on what was missing from their care that would be beneficial to others if it were available. 

### Qualitative Analysis

Verbatim written responses to open-ended questions were entered into an Excel spreadsheet and a conventional content/theme analysis was conducted [24,25]. The analysis was separately completed for each question without imposing preconceived notions about categories. Both the content and the frequency of topics mentioned were captured. Two team members, both of whom had extensive qualitative training and experience and worked in oncology settings, designed the coding framework. Each independently read the written comments and identified the content topics within the responses. Similar topics were collated, and the content labels were derived from the respondent data. The researchers discussed their independent observations and arrived at a consensus about the coding categories. This set of categories was then used to code the data. The third author reviewed the analysis. 

All responses were coded by two team members and any disagreements were discussed to achieve consensus. The content of each category was reviewed by both team members and categories were grouped into broader conceptual domains. Coded responses were grouped by age to explore if there were different perspectives being shared. For this analysis, adolescent and Young Adults (AYA) were defined as between 18 and 29 years; adults as between 30 and 64 years; and older adults as 65 years and older. Given that each age group may have different options for generating income and accessing savings, perspectives may vary in terms of the impact of financial burden. The results for those respondents who did not disclose their ages were not included in this analysis.

## 3. Results

### 3.1. Demographic Characteristics

A total of 1708 survey respondents who reported their income indicated a low annual household income (<$25,000 CD); 1008 (59%) were female, 685 (41.9%) were male, and 15 (0.9%) preferred not to answer (Table 1). A total of 55 respondents were aged 18 to 29 years old (3.2%); 445 (36.1%) were 30 to 64 years old; and 1198 (70.1%) older adults were 65 years old or older. Ten respondents preferred not to disclose their age and were not included in the analysis. The majority, 1331 (77.9%), reported their education levels as being at high school or lower levels. 

### 3.2. Perspectives from Written Comments

The written comments from respondents ranged in length and depth. Many simply wrote a word (e.g., “*pain*”, “*fatigue*”), while others wrote a phrase (“*recovery took a long time*”) or several sentences. Often, respondents described several topics within their written comments to one question. The reporting below focuses on the responses to each of the three open-ended questions.

### 3.3. Major Challenges

There were 1782 major challenges identified, with about two-thirds (60.7%) focused on issues regarding physical concerns (e.g., capacity (fatigue, mobility issues, weakness), pain, numbness, swelling, and other effects of therapy (long recovery, comorbidities, infection) (Table 2). Over half of the adults and older adults in this low household income group commented on fatigue and loss of energy, and close to a third (30.0%) reported mobility restrictions and limitations. Comments included: “*I have never felt that kind of fatigue before”* and an inability *“to do things that I used to be able to do*”. Nearly seven out of ten respondents (68.8%) who commented on pain, numbness, or swelling reported a lack of pain management: “*The pain I had after treatment in all my bones and joints*” commented one respondent, “*made it hard to stand*”.

Fewer respondents reported emotional and practical concerns as the major challenges following the end of treatment. Emotional concerns (e.g., coping, fears, depression, anxiety) were reported by 259 (14.5%) respondents, 119 (6.7%) reported practical concerns (e.g., requiring help with chores/errands, transitioning back to work/school, financial issues), and 117 (6.6%) commented on the challenge of making lifestyle adjustments (e.g., returning to normal, difficulty eating and/or sleeping). Examples of comments illustrated the intersection of the physical, emotional, and practical: ”going back to work, it took longer than I expected to get back in the swing of things”, “coming to terms with my limitations, dealing with residual pain from overactive nerves”, and “unable to look after my house and year and unable to return to normal everyday duties of life”.

Other comments about the major challenges experienced focused on difficulties with service delivery (e.g., cancer centers, hospitals, and healthcare providers), relationships/support concerning family or friends, and lack of emotional support in general. For example, one respondent wrote: “there was no support telling me how I can receive financial aid, emotional aid, etc.”. While adults aged 30 to 64 years comprised 26% of the total respondents, 34.7% of the comments on major challenges were submitted by this group; 60.7% were submitted by older adults.

### 3.4. Positive Experiences

There were 1171 positive experiences reported (Table 3). Together, the majority of the responses focused on the value of having emotional and practical support available (293, 25.0%), self-care strategies and advice respondents would offer to others (272, 23.2%), and appreciation for having knowledgeable and attentive healthcare providers (266, 22.7%). Having support from family and friends was cited by many respondents as among the top positive experiences, as were the things they did for themselves to remain feeling optimistic. Many comments highlighted the importance of staying positive during the cancer experience and not allowing the disease to overwhelm them. For example: “I’ve learned to ask questions—I do not assume or self-diagnose”; “take things one day at a time; don’t try to overanalyze things”; “keep your appointments and let the doctors do their job”; and “cancer is not a sentence, it is only a diagnosis”. 

Other positive experiences included access to and existence of timely follow-up care; clear, timely, and ease of communication with healthcare providers; and appreciation for successful treatment and good cancer treatment experiences overall. These survivors valued the timely access they had to diagnosis, treatment, and follow-up care as well as healthcare professionals who were knowledgeable and compassionate. “When I go back for check ups with my surgeon at the hospital, the nurses come see me and give me a hug and we chat”, wrote one respondent, “just nice to be remembered and makes it feel like a real “team” approach to my care”. Less than 5.0% of all respondents indicated they experienced nothing positive during their survivorship care. There was also a sense among the comments of the need for self-reliance. For example, one respondent wrote: “You cannot depend on anyone else to help you; so you learn inner strength and resilience”. Comments from older adults were 63.7% of the total responses about positive experiences.

### 3.5. Suggestions for Improvement

The majority of the 1140 suggestions for improvement highlighted the need for better emotional, practical, and other support (297, 26.1%); improved information about self-care/cancer prevention, side effect management and programs/services (236, 20.7%); and greater access to and provision of timely, regular follow-up care and testing for recurrence (213, 18.7%) (Table 4). In addition, about 10% (109, 9.6%) of comments were written by adult and older adult respondents for this question, which simply reflected a positive statement. These comments included: “I survived cancer” and commonly referred to “excellent/good care”, “satisfying experience”, and appreciation of care by “excellent healthcare professionals”. One hundred and eight respondents offered no suggestions for improvement (9.5%). Furthermore, 60 (5.3%) of the suggestions were made by AYA respondents, 412 (36.1%) by adults and 668 (58.6%) by older adults. 

Additional supportive services were most commonly identified to address practical concerns. These included services that would assist with financial aid, return to work/school, and help with chores and transportation. As examples, survivors wrote: “I am attempting to repay my debts, although I have not yet found permanent employment”, “cancer patients should have a benefit supplement every month (income)”, and “there should be a separate financial assistance program dealing solely with cancer patients”. 

The need for peer support, self-help, and information about support was frequently mentioned. Survivors thought it was important to have detailed information about survivorship care plans, available services, management of post-treatment issues, and what to expect following treatment. For example, respondents wrote, “an easier way to communicate with cancer doctors about small questions post-treatment: having a place to ask questions is so important”; “lack of information concerning funding for trips, appointments, and medications”; and “when all treatments were done…it was just like…ok…you are cancer free now, see ya. I had no tools to help me”.

The suggestions survivors provided focused on the need for timely and regular follow-up care and appointments, access to caring health care professionals, and guidance about post-treatment therapies. Guidance and clear information were seen as reducing uncertainty. Examples of comments written by respondents included: “more careful monitoring for recurrence in follow-up with a surgeon”; “I was told I am not ‘cancer free’ for 10 years; I would like to have a connection with my oncologist yearly for 10 years”; and “it would help if a doctor or a nurse would tell what is going on, and what should be done or, what I should be doing”.

### 3.6. Comparison across Age Groups

A comparison of perspectives across age groups revealed similarities in both the types and frequency of comments from each group. Major challenges were most frequently identified as physical in all groups. The most frequent positive experiences surrounded the support from family, friends, and peer support. The top practical suggestions for improvement were related to programs for financial aid, return to work or school, and assistance with transportation. Some understandable differences were apparent, for example, proportionally more older adults were concerned with the need for help with chores and travel than transitioning back to work compared to adult respondents. Young adults and adults generally sought improved financial support, longer unemployment benefits/sick time, and better health coverage.

## 4. Discussion

A secondary analysis was conducted to explore the perspectives of cancer survivors living with an annual household income of <$25,000 CD regarding their major concerns, what they found positive about their cancer care, and suggestions for improvement. The analysis drew from written responses to a national survey provided by respondents. The analysis provides an opportunity to increase our understanding about the type of challenges confronting individuals with low household income during the early survivorship period from their points of view. It also allows insight into the similarities in terms of perspectives emerging within different age groups regarding these issues. The analysis was conceptualized as exploratory, given that little work has been undertaken regarding low-income cancer survivors. 

The sample contained individuals from across the country living within various provincial healthcare systems with different policies. However, it is important to note that the perspectives from the older age group (65 years and above) dominated. It is also noteworthy that some older adults reported low household annual income, but had access to assets such as retirement and/or investment funds that would help alleviate the financial impact of cancer. 

Many of the perspectives described by this sample of survivors mirrored perspectives identified in other cancer survivor reports [26,27,28,29]. A cross-section of challenges were identified, including physical, emotional, and practical issues. A lack of information and what these survivors saw as inadequate follow-up care was experienced by many. Follow-up care was frequently described as limited, non-existent, and/or not helpful. Some felt abandoned by the system when primary cancer treatment finished and there was uncertainty about future plans for their care. 

At the onset of the analysis, it was anticipated there would be frequent mention of financial issues within the perspectives of this low-income sample. However, this was not evident. While many wrote about the need for financial aid as an area needing improvement, financial challenges overall did not emerge as the predominant issue for most respondents in this sample. Research has shown that Canadians pay 30% of cancer-related costs, but the burden is highest in the year post diagnosis [28]. Given two-thirds of respondents were more than a year post treatment, when funds for travel to frequent clinic appointments and possible equipment and other expenses were no longer required, financial aid may no longer have been an overriding concern. 

However, those who did write about financial concerns described difficult challenges in accessing both government supplements and insurance benefits, understanding how they worked, and how to apply. Many mentioned the need for ongoing financial assistance programs and/or information about such programs to cover medications, equipment, and home care. Suggestions for improvement included free or low-cost programs and therapies that currently require fees or have limited coverage. It is not clear to what extent respondents were aware or informed of existing aid programs and services that were potentially available. One wonders if an in-person interview approach would have generated more direct comments about specific financial challenges and available/accessible solutions. 

Physical issues were identified most frequently as the major challenges for these survivors, focusing on debilitating issues such as fatigue/exhaustion, mobility challenges, pain management, and other therapy effects, such as long recovery times, and comorbidities. This aligns with other reports from cancer survivors [5,26,29,30]. Physical effects can prevent or limit the ability of survivors to find, stay at, and/or return to work, and thus, earn income, especially in some types of occupations. Additionally, many low-income earners have jobs that require a high physical labor component. In particular, older adults may have added physical burdens from co-morbidities that increase the challenges they experience returning to pre-cancer levels of physical function. Many of the suggestions for improvements from these survivors were aligned with the need for post-therapy programs, which included physiotherapy/rehabilitation and assistance in returning to work. These types of rehabilitation programs may require payment over the long term and may not be available in rural areas. Additionally, survivors who have been discharged from cancer care may not have access to family doctors or nurse practitioners to assist with these physical effects or access to such programs. 

In terms of positive experiences, what stood out in these data was the value of support from family and friends and the reliance on oneself to remain positive and strong during what many regard as a difficult and challenging time. Cancer patients have identified the importance of support, both emotional and practical, from family and friends in previous reports [31,32]. However, the importance of maintaining a positive attitude and focusing on self-care offered insight into how patients and survivors cope with this situation, especially those in the low-income bracket. These perspectives align with research on topics regarding the use of positive coping strategies to deal with stressful situations [33,34,35,36], the importance of resilience in low-income populations [37], and the reasons why some cancer survivors elect not to seek help [38]. 

The suggestions for improvements during the survivorship period were aligned with the desire for programs or assistance with self-care and emotional support, as well as providing information that would facilitate individuals taking action in terms of their own care. What survivors saw as positive or helpful, or what would have been helpful to them if it had been offered, was the basis for suggestions about improving the cancer system. Access to and information about available programs following the completion of cancer treatment and programs that offered assistance in coping, dealing with anxiety/depression, and other emotional issues were frequently cited. Existing programs frequently have long wait times and fees, or only offer limited “free” service at present across the country.

### 4.1. Limitations

Several limitations exist with this analysis. Confidentiality issues limited information about survivors that could be shared from the registry, leaving insufficient details to allow the weighting of survey results and to determine their representation of all Canadian survivors of cancer. Over 23.0% of respondents did not disclose their annual household income in the original Transitions Study, and there was no way to assess whether the missing data were random over the income groups. The Transition Study sample does not reflect the income distribution across Canada [39]. Low-income populations and non-English/French speakers are underrepresented. Hence, results from this secondary analysis cannot be generalized to the Canadian population with low annual household income.

This secondary analysis was an exploration using a publicly available data set, thus imposing limitations on the variables available for incorporation into this work. Future analysis would benefit from including educational and occupational data and incorporating other social determinants of health. Additionally, the dataset was established in 2016, and there have been increasing financial challenges and healthcare access difficulties in Canada during and following the COVID-19 pandemic. Perspectives of cancer survivors living in low-income circumstances now may differ from those reported in this paper.

Finally, the measure of income was objective, asking about annual household income, and may not directly correspond to or reflect perceived financial difficulty. For example, older adults could have considerable savings or investments, yet low household income. It also does not account for the number of people in the household or whether the amount identified in the survey was a decrease from before the cancer diagnosis. Perceived financial difficulty may also be associated with the region in which one lives, as the cost of living may vary by geographic region, as does the availability and costs of specific healthcare services. Measuring perspectives regarding financial toxicity would also extend the understanding of the impact on individuals. 

### 4.2. Implications

The results of this analysis emphasize the importance of healthcare professionals being aware of the potential challenges for survivors, especially those in low-income brackets. Clinicians ought to conduct assessments of risk for experiencing financial difficulties with patients/survivors and hold intentional conversations about the possible impact of financial issues. There is also a need to consider programs or referrals to enhance resilience and positive coping. Cancer centers may also consider the provision of information regarding available financial support/programming as part of standard care practices.

## 5. Conclusions

The results of this analysis emphasize the need for cancer programs and policymakers to explore financial support programming. Given the escalating cost of living following the worldwide pandemic, and the potential impact of financial difficulties on the quality, level, and speed of recovery for survivors, new initiatives may be required. 

## Figures and Tables

**Table 1:** Respondent Profile (n = 1708).

Variable	Number	Percentage
**Sex**		
Male	685	40.1%
Female	1008	59.0%
No answer	15	0.9%
**Age**		
18–29	55	3.2%
30–64	445	26.1%
65 and older	1198	70.1%
No answer	10	0.6%
**Marital Status**		
Single	254	14.9%
Married/partnered	658	38.5%
Separated/divorced/widowed	768	45.0%
Prefer not to answer	28	1.6%
**Education**		
High school or less	1331	77.9%
Post-secondary degree (college/university)	276	16.2%
University graduate degree	37	2.2%
Missing	64	3.7%
Disease site *		
Prostate	297	17.4%
Colorectal	421	24.7%
Breast	507	29.7%
Melanoma	126	7.4%
Hematological	180	10.5%
Other	114	6.7%
Missing	122	7.1%
**Metastases**		
No metastases	1164	68.1%
Living with metastases	180	10.5%
Unsure	233	13.6%
Missing	131	7.7%
**Time since treatment**		
<1 year	197	11.5%
1 year to <3 years	745	43.6%
3 years or more	410	24.0%
Did not receive treatment	278	16.3%
Missing	78	4.6%
**General physical health (one item)**		
Very poor/poor	152	8.9%
Fair	565	33.1%
Good/very good	984	56.7%
Missing	7	0.4%
**General emotional health (one item)**		
Very poor/poor	129	7.6%
Fair	416	24.4%
Good/ very good	1041	60.9%
Missing	122	7.1%
**Overall quality of life (one item)**		
Very poor/poor	85	5.0%
Fair	497	29.1%
Good/very good	1118	65.5%
Missing	8	0.5%
**Comorbidities**		
Yes	1201	70.3%
No	427	25.0%
Missing	80	4.7%
**Comorbidities (four most common)**		
Cardiovascular or heart condition; hypertension or high blood pressure	544	45.3%
Arthritis, osteoarthritis, or other rheumatic disease	600	50.0%
Diabetes	271	22.6%
Mental health issues	258	21.5%

* Percentages add to greater than 100 because respondents could select multiple sites.

**Table 2:** Major challenges identified.

Major Challenges n = 1785			Number of Comments by Age Groups			Topic Total	Percentage of Topic
Categories	Number	Sub-Topics	AYA 18–29 N = 82	Adults 30–64 N = 619	Older Adults 65+ N = 1081		
**PHYSICAL**	**1085** (60.8%)	Capacity (fatigue/mobility)	9	139	246	**394**	36.3%
		Pain/Numbness/Swelling	3	61	96	**160**	14.7%
		Other Side Effects *	7	44	84	**135**	12.4%
		Therapy Effects **	1	34	62	**97**	8.9%
		Bowel Problems	1	17	73	**91**	8.4%
		Urological Effects	0	12	39	**51**	4.7%
		Body Image	6	18	21	**45**	4.1%
		Post-Surgical Complications	3	12	25	**40**	3.7%
		Sexual/Fertility Concerns	4	15	21	**40**	3.7%
		Cognitive Effects	4	15	13	**32**	2.9%
**EMOTIONAL**	**259** (14.5%)	Emotional issues, coping ***	5	31	54	**90**	34.7%
		Fears (recurrence/death)	5	29	40	**74**	28.6%
		Depression/Anxiety	7	34	31	**72**	27.8%
		Stress	4	8	11	**23**	8.9%
**PRACTICAL**	**119** (6.7%)	Chores/transportation help	0	12	40	**52**	43.7%
		Return to work/school	5	27	6	**38**	31.9%
		Financial concerns	5	13	11	**29**	24.4%
**LIFESTYLE ADJUSTMENTS**	**117** (6.6%)	Returning to normal	5	25	45	**75**	64.1%
		Difficulty eating	2	11	20	**33**	28.2%
		Difficulty sleeping	1	3	5	**9**	7.7%
**SERVICE DELIVERY**	**92** (5.2%)	Information/Communication	0	8	24	**32**	34.8%
		Follow-up Care	1	10	18	**29**	31.5%
		Hospital/Clinic Services	1	8	11	**20**	21.7%
		Healthcare Providers	0	6	5	**11**	12.0%
**RELATIONSHIPS, SUPPORT**	**51** (2.9%)	Family challenges/concerns	2	12	14	**28**	54.9%
		Lack of emotional support	0	7	9	**16**	31.4%
		Challenges with friends	0	2	5	**7**	13.7%
**NO CHALLENGES**	**26** (1.5%)	No challenges reported	1	4	21	**26**	*100.0%*
**OTHER**	**24** (1.3%)	No or still in treatment	0	2	22	**24**	*100.0%*
**POSITIVE**	**12** (0.7%)	Positive comments	0	0	12	**12**	*100.0%*

* Other Side Effects of Therapy; examples: long recovery, comorbidities, infection, weight gain/loss. ** Therapy Effects; medication, chemotherapy, radiation effects. *** Emotional Issues/Coping; examples: anger, low self-esteem/motivation, insecurity, vulnerabilities.

**Table 3:** Positive experiences.

Positive Experiences n = 1171			Number of Comments by Age Groups			Topic Total	Percentage of Topic
Categories	Number	Sub-Topics	AYA 18–29 N = 52	Adults 30–64 N = 373	Older Adults 65+ N = 746		
**SUPPORT**	**293** (25.0%)	Support from family and friends	5	29	134	168	*57.3%*
		Peer and group support	4	25	31	60	*20.5%*
		Help from others/HCPs	1	17	28	46	*15.7%*
		Faith/spiritual support	1	1	9	11	*3.8%*
		Practical support	1	4	3	8	*2.7%*
**SELF-CARE/ADVICE TO OTHERS**	**272** (23.2%)	Stay positive, confident	3	31	70	104	*38.2%*
		Ask for help/trust HCPs	0	22	44	66	*24.3%*
		Have faith/live each day	1	15	23	39	*14.3%*
		Other (stay calm/healthy)	8	16	39	63	*23.2%*
**HEALTHCARE PROVIDERS**	**266** (22.7%)	Excellent/knowledgeable HCPs	7	54	59	120	*45.1%*
		Support from HCPs, cancer centre	8	30	33	71	*26.7%*
		Attentive, compassionate, caring HCPs	1	19	33	53	*19.9%*
		Good access to HCPs, specialists	0	9	13	22	*8.3%*
**FOLLOW-UP CARE**	**90** (7.7%)	Regular/timely follow-up	0	12	22	34	*37.8%*
		Care by doctors, oncologists, surgeons	1	11	22	34	*37.8%*
		Routine tests/home visits	0	5	17	22	*24.4%*
**INFORMATION AND COMMUNICATION**	**60** (5.1%)	Good communication with HCPs	1	8	27	36	*60.0%*
		Good information/answers to questions	1	8	15	24	*40.0%*
**CANCER CENTRE**	**14** (1.2%)	Good experience at centre	2	4	8	14	*100.0%*
**COMPLIMENTARY/SUPPORT THERAPIES**	**12** (1.0%)	Examples: meditation, yoga, naturopathy, art	1	7	4	12	*100.0%*
**POSITIVE**	**76** (6.5%)	Successful treatment	2	16	27	45	*59.2%*
		Good experience, other	3	8	20	31	*40.8%*
**NO POSITIVE EXPERIENCES**	**51** (4.4%)	Nothing positive to report	1	14	36	51	*100.0%*
**OTHER**	**37** (3.2%)	No follow-up care required	0	4	23	27	*73.0%*
		Other (still in treatment)	0	4	6	10	*27.0%*

**Table 4:** Suggestions for improvements.

Suggested Improvements n = 1140			Number of Comments by Age Groups			Topic Total	Percentage of Topic
Categories	Number	Sub-Topics	AYA 18–29 N = 60	Adults 30–64 N = 412	Older Adults 65+ N = 668		
**SUPPORT/SELF-CARE**	**297** (26.1%)	**Practical**				**131**	*44.1%*
		Financial aid	5	42	16	63	
		Help with chores/travel	2	11	24	37	
		Return to work issues	5	11	15	31	
		**General**				**94**	*31.6%*
		Services/groups (plus peer)	5	23	31	59	
		Family/friends support	1	11	18	30	
		Other	0	2	3	5	
		**Emotional**				**52**	*17.5%*
		Personal/one on one	1	14	14	29	
		Help with issues, coping	2	12	9	23	
		**SELF-CARE**				**20**	*6.7%*
		Be your own advocate	1	4	6	11	
		Other (faith, rest, healing)	0	3	6	9	
**INFORMATION AND COMMUNICATIONS**	**236** (20.7%)	**Information**				**189**	*80.1%*
		Self-care, recurrence, care plans, Other	6	35	39	80	
		Side effects/post-treatment issues	2	30	42	74	
		Programs/services/support groups	4	12	19	35	
		**Communication**				**47**	*19.9%*
		With/among HCPs	0	20	27	47	
**FOLLOW-UP CARE**	**213** (18.7%)	Timely/regular/care	6	39	64	109	*51.2%*
		Access to/care by HCPs	0	13	36	49	*23.0%*
		Post-treatment therapies	14	14	12	40	*18.8%*
		Other	1	1	13	15	*7.0%*
**HEALTHCARE PROVIDERS**	**90** (7.9%)	Attentive, compassionate HCPs	0	18	23	41	*45.6%*
		Good/knowledgeable HCPs	0	14	21	35	*38.9%*
		Other (better access)	1	4	9	14	*15.6%*
**CLINICS/HOSPITAL SERVICES**	**70** (6.1%)	Improved services/facilities/closer to home	1	7	26	34	*48.6%*
		Shorter wait times for results/appointments	3	12	16	31	*44.3%*
		Other (e.g., address patient preferences)	0	4	1	5	*7.1%*
**POSITIVE COMMENTS**	**109** (9.6%)	Great care, satisfying experience	0	24	56	80	*73.4%*
		No concerns, needs met	0	4	25	29	*26.6%*
**NO SUGGESTIONS**	**108** (9.5%)	No suggestions	0	27	81	108	*100.0%*
**NEGATIVE COMMENTS**	**17** (1.5%)	Negative comments	0	1	16	17	*100.0%*

## Data Availability

Canadian Partnership Against Cancer has full control of primary unidentifiable record level data. The dataset is publicly available (https://www.systemperformance.ca/transition-study/, (accessed on 12 March 2022).

## Appendix A

**Table:**

Ethics Boards	Approval	Number
University of Saskatchewan Behavioral Research Ethics Board	12 April 2016	BEH # 16-79
Comité d’éthique de l’Institut de la statistique du Québec	4 March 2016	Approved by title
PEI Research Ethics Board	17 March 2016	Approved by title
Ontario—Hamilton Integrated Research Ethics Board	5 April 2016	#1528
Newfoundland and Labrador Health Research Ethics Board	24 March 2016	#20216.080
University of Manitoba—Health Research Ethics Board	21 March 2016	HS19571(H2016.114)
Health Research Ethics Board of Alberta—Cancer Committee	1 April 2016	HREBA.CC-16-0025
Nova Scotia Health Authority Research Ethics Board	11 April 2016	#1021104
New Brunswick—Corporate Privacy Office—Department of Health	25 May 2016	Approved by title
British Columbia—Provincial Research Ethics Board	May 2016	Approved by title
